# RETRACTION: Epoetin Alpha and Epoetin Zeta: A Comparative Study on Stimulation of Angiogenesis and Wound Repair in an Experimental Model of Burn Injury

**DOI:** 10.1155/bmri/9759817

**Published:** 2026-04-24

**Authors:** BioMed Research International

RETRACTION: N. Irrera, A. Bitto, G. Pizzino, M. Vaccaro, F. Squadrito, M. Galeano, F. Stagno d’Alcontres, F. Stagno d’Alcontres, M. Buemi, L. Minutoli, M. R. Colonna, D. Altavilla, “Epoetin Alpha and Epoetin Zeta: A Comparative Study on Stimulation of Angiogenesis and Wound Repair in an Experimental Model of Burn Injury,” *BioMed Research International*, 2015, 968927, https://doi.org/10.1155/2015/968927

The above article, published online on 04 June 2015 in Wiley Online Library (wileyonlinelibrary.com), has been retracted by agreement between the journal Editor‐in‐Chief, Yoel Klug; and John Wiley & Sons Ltd. The retraction has been agreed due to concerns identified with the figures [1, 2]. Specifically:

• The first and second B‐actin band shown in Figure 3c appear identical
• In Figure 3a, the 5th and 6th VEGF bands appear identical to the 7th and 8th VEGF bands
• In Figure 3a, the 4th VEGF band appears similar to the 2nd CDK6 band in Figure 3c after 180 degree rotation and horizonal flip
• In Figure 3a, all of the *β*‐actin bands appear identical
• In Figure 3c, the 3rd and 5th CDK6 bands appear identical
• In Figure 3c, the 1st and 8th CDK6 bands appear similar when rotated 180 degrees and horizontally stretched
• In Figure 5a, the 1st and 2nd Cyclin E bands appear identical to the 8th and 9th Cyclin E bands
• In Figure 5b, the 6th and 7th *β*‐actin bands appear identical to the 8th and 9th *β*‐actin bands

Additionally, ethics concerns have been raised regarding the treatment of the animals used in the study [2], and the authors could not provide the ethical approval documentation.

After assessment of the concerns and the author’s response, the editorial board have confirmed the retraction of the article due to loss of confidence in the reliability of the results and ethics concerns.

The authors did not respond to the retraction.
